# A comparison of reading times in full-field digital mammography and digital breast tomosynthesis

**DOI:** 10.1186/bcr3281

**Published:** 2012-11-09

**Authors:** SJ Connor, YY Lim, C Tate, H Entwistle, J Morris, S Whiteside, J Sergeant, M Wilson, U Beetles, C Boggis, F Gilbert, S Astley

**Affiliations:** 1 Manchester Medical School, University of Manchester, UK; 2 Nightingale Breast Centre, University Hospital of South Manchester, Manchester, UK; 3 Institute of Population Health, University of Manchester, UK; 4 Department of Medical Statistics, University of Manchester, UK; 5 School of Clinical Medicine, University of Cambridge, UK

## Introduction

Currently, breast screening is implemented with X-ray mammography, but new technology such as digital breast tomosynthesis (DBT) may provide significant advantages because it produces three-dimensional breast images with high spatial resolution. To aid assessment of the cost-effectiveness of DBT, we have compared reading times for DBT images and full-field digital mammograms (FFDM) of the same women.

## Methods

Four consultant radiologists were timed by stopwatch reading FFDM images and then DBT images, with a total of 119 timings of two view cases for each imaging modality. The mammograms used were from women recalled following a screening mammogram and from women who are screened annually due to a family history of breast cancer. The time taken to display the images and to report on them was excluded from the analysis.

## Results

The reading time was significantly longer for DBT images than for FFDM images, with median times of 66 seconds and 17 seconds, respectively (*P *< 0.001), with times for FFDM ranging from 4.7 to 109.0 seconds and for DBT from 31.9 seconds to 180.6 seconds. The ratio of reading times DBT:FFDM was approximately 4:1, with readers varying between ratios of 2.4 to 4.9.

## Conclusion

Reading times were greater for DBT as expected, since the reader has to scroll through the images slices to visualise the entire breast. The images used in this study were likely to have a higher rate of abnormalities than screening mammograms, and to have higher breast density, both of which will increase reading time over routine screening.

